# “You Have to Go Gently”: Mentors’ Perspectives of a Peer Mentoring Empowerment Program to Reduce Marginalization in Refugee and Migrant Women

**DOI:** 10.3390/ijerph19116434

**Published:** 2022-05-25

**Authors:** Shelley Gower, Zakia Jeemi, Jaya A. R. Dantas

**Affiliations:** 1 Curtin School of Nursing, Curtin University, Perth 6102, Australia; shelley.gower@curtin.edu.au; 2 Curtin School of Population Health, Curtin University, Perth 6102, Australia; zakia.jeemi@curtin.edu.au

**Keywords:** peer-mentoring, refugee, migrant, women, empowerment, employability, co-design

## Abstract

The Empowerment and Peer Mentoring of Migrant and Refugee Women pilot program (EMPOWER) provides a mechanism for migrant women who have established lives in Australia to mentor newly arrived women to build the ability, confidence, and knowledge to overcome barriers to the social determinants of health such as employment. Female migrant mentors (n = 21) met with their mentees (n = 32) on a regular basis over a period of 3 to 12 months between September 2019 and November 2021. The individual mentoring was augmented by group workshops facilitated by content experts and the research team. The unique perspectives of the mentors were explored through individual interviews (n = 15) and analysis of journal entries (n = 58) submitted regularly by mentors throughout the program. Thematic analysis revealed that mentors were intrinsically motivated to build strong and trusting connections with their mentees, which were pivotal to reducing inequalities for mentees and their families. Mentors had high expectations of themselves and demonstrated commitment and flexibility to accommodate mentees’ needs. However, they sometimes struggled when supporting mentees who were overwhelmed by the systemic and other stressors associated with resettlement and pre-migration trauma. Regular networking and moral support for mentors would enhance future programs.

## 1. Introduction

Since the end of 2019, 79.5 million people have been forced to leave their homes due to civil conflict, persecution, human rights violations and environmental or economic pressures [1]. In addition to this, skilled migrants have moved to other countries to pursue employment opportunities, placing global migration numbers at 281 million people in 2020 [2].

Australia’s Refugee and Humanitarian program offers resettlement to people who have been found to be in need of refuge according to the Refugee Convention [3]. The annual intake numbers fluctuate according to global events. For example, in 2020 the Australian Federal Government reduced its annual humanitarian intake from 18,750 to 13,750 due to economic pressures caused by the coronavirus disease (COVID-19) outbreak, but in March 2022 agreed to increase the number of temporary humanitarian visas to accommodate people fleeing the Ukraine conflict [4]. During the COVID-19 pandemic, skilled migration to Australia was halted, but between 2022 and 2023, to boost Australia’s economy, the Migration Program will accept 109,900 skilled migrants and their families [5].

### 1.1. Migration Stress

Migration is a known stressor, especially for people displaced by conflict who may experience health impacts that include emotional and physical trauma associated with pre-migration contexts [6,7]. They may be isolated from their families, lose social capital through spending time in refugee camps and have a lack of access to the social determinants of health such as education [8,9]. Skilled migrants make a conscious decision to move to another country but may still face cultural dissonance and isolation. Unskilled female refugees and migrants may experience greater isolation due to language difficulties, a potential lack of formal qualifications and cultural expectations around family responsibilities [10,11].

### 1.2. Employment and Health

The Australian government considers resettlement to be successful when people are financially independent and are contributing to the economy [12]. Employment is a priority for newly arrived refugee and migrant families [13] and enhances migrants’ sense of belonging [14,15]. It is also a known social determinant of health, the lack of which leads to lower access to resources, less social inclusion, and poorer mental health [9].

However, despite having the right to work [16], refugees and migrants encounter language difficulties, racism, and non-recognition of skills, among other barriers, when seeking employment [17,18,19]. Refugee and migrant women face particular barriers when seeking employment such as a reluctance to use formal child care [17], a lack of networks and experience, employer attitudes towards cultural dress [10,11,20] and a bias towards males’ access to employment and financial independence [21]. This occurs not only in Australia but globally [22].

### 1.3. Peer Mentoring

Peer mentoring is a process by which an individual with experience and skills shares their knowledge and supports and encourages a mentee seeking to further their development and agency in a similar area [23]. Peer mentoring can result in mutual benefits for both mentors and mentees, leading to personal growth and capacity building [23]. Peer mentoring programs have been used with culturally and linguistically diverse (CALD) populations in migration contexts to promote inclusion, a sense of belonging and enhance access to the social determinants of health [24,25]. Peer-led programs are culturally safe and draw on the principles of social justice, access and equity [26]. Participating in mutually supportive relationships appears to be beneficial for refugee women, enhancing social connection and providing validation of their migration experiences [27,28].

Most evaluations of peer mentoring programs for refugee and migrant women have focused on the health and social outcomes for the mentees [27,29,30,31,32,33]. The health and well-being outcomes of participants in the current study have been previously published [34]. There is evidence that these programs result in an increase in confidence, self-efficacy, and social connection [34]. However, given the critical and intimate role of the mentors in the process, their perspectives can provide unique insight into how peer mentoring programs can be further developed and refined for future cohorts. Studies to date that have explored mentors’ perspectives have been undertaken in programs with undergraduate students [35,36,37,38] and early career teachers [39]. Only one study explored mentors’ experiences in a community-based mentoring program supporting refugees [40]. Findings showed an increase in mentor resilience and empowerment, aligning with the principle of mutual benefit in the mentor-mentee relationship.

The Empowerment and Peer Mentoring of Migrant and Refugee Women pilot program (EMPOWER) was developed to provide opportunities for migrant women who have settled in Australia to share their knowledge and experiences to support newly arrived migrant and refugee women in a holistic way, building empowerment, improving well-being and enhancing their access to the social determinants of health such as employment. This article reports the findings of the process evaluation of this co-designed, pilot participatory program, with a focus on the mentors’ experiences and their resulting recommendations.

## 2. Materials and Methods

A community-based participatory approach (CBPA) was used to develop and facilitate the EMPOWER program. Input from potential mentees and mentors, our community partners and Western Australian organizations that provide support to CALD communities guided the development of the content of the program.

### 2.1. Development of the Mentoring Program and Program Content

A mixed methods community assessment using focus group discussions and questionnaires was undertaken with 34 refugee and migrant women affiliated with our primary community partner, Ishar Multicultural Women’s Health Service. The content and focus of the mentoring program were informed by the current gaps, expectations, needs, knowledge, and skills of refugee and migrant women through the community assessment. Feedback and input from potential mentors, key stakeholders from the supporting community organizations and representatives from local government also informed the development of the program.

The EMPOWER pilot program was designed to provide refugee and migrant women with a program that was holistic and culturally informed. The program was designed to develop social capital and concepts of community participation and a sense of belonging, links to community groups and resources, emotional and social support. The anticipated outcomes were an improvement in employment skills, reduced isolation, and improvements in overall health and well-being. The mentors were provided with a set of guiding topics as part of their training which they covered with their mentees during peer mentoring sessions. Although guiding topic areas were provided, mentors had the discretion to tailor the discussions and support provided to areas of most relevance to the mentee. Mentors were also asked to follow a code of conduct that stipulated that mentors were not to assume a role of advocacy on behalf of their mentees and were not to have expectations of mentees outside the scope of the program such as those that might be expected of an employee. All mentors were required to treat their mentees with dignity, respect, sensitivity and in a non-discriminatory manner at all times.

Individual mentoring sessions were augmented by group workshops that covered topics relating to job-readiness and employment were developed and delivered by the researchers and associated providers. In keeping with the flexible approach of community-based participatory research, workshops were adjusted to suit the needs of each cohort of mentees.

The final program format consisted of individual mentoring sessions approximately twice per month and group workshops and was delivered between September 2019 and November 2021, lasting between 3 and 12 months across five groups of mentors and mentees. Each cohort was recruited through different community partners. Eleven group workshops covering topics on English for Employment, Employment Skills (job applications and job interview skills), and Financial Management and Starting a New Business were delivered by content experts to mentees throughout the program. Mentors were also encouraged to attend these workshops. Further information about the development and the content of the program, the participant cohorts, the workshops, data collection methods, and outcomes for mentees is published by Gower et al. (2022) [34].

### 2.2. Participant Recruitment and Matching Mentors with Mentees

Through purposive and snowball sampling, the researchers, with the help of the community partners, helped identify potential mentors from their network of service providers that provide support to refugee and migrant women in the Western Australian community. Inclusion criteria for mentors were female migrants who had established careers in the Australian workforce and were committed to meet with a mentee approximately twice per month for mentoring sessions. A total of 21 mentors were recruited and trained by the research team through a 3 h mentor training program [34].

The inclusion criteria for mentees were initially refugee or humanitarian entrants, however, through discussions with stakeholders and community partners, and in keeping with the CBPA, migrant women from non-humanitarian backgrounds with limited English and employability skills were identified as a similar group in need and therefore, also included in the inclusion criteria. The final inclusion criteria for mentees were refugee or humanitarian entrants and migrant women from non-humanitarian backgrounds with limited English and employability. A total of 21 mentors participated in the program along with 32 mentees across five cohorts.

Where possible, mentors and mentees were matched according to prior areas of work, if any, or area of employment interest, education background, and geographical location. Initially, mentors and mentees who spoke different languages were matched to encourage English conversation. However, when some participants withdrew or voiced difficulty in communicating with their mentor or mentee, some rearrangement of the pairings was required. Consequently, the decision to keep language groups separate was changed.

### 2.3. Ethical Considerations

Ethical approval was obtained from the Curtin University Human Research Ethics Committee (HRE2018-0310). Participants provided written, informed consent to participate in the study and for their data to be used in the research. Mentors and mentees were provided with a code of ethics developed in consultation with community partners to guide conduct and behavior and to facilitate a welcoming, non-judgmental and respectful peer mentoring environment.

### 2.4. Interruptions Due to COVID-19

In March 2020, the COVID-19 pandemic impacted Western Australia, with lockdowns and social distancing measures imposed across Australia [41]. With significant job losses across Australia due to the closure of businesses during the pandemic, seeking employment became particularly difficult for refugee and migrant women. Furthermore, they were isolated and often carried domestic burdens such as home-schooling children. Consequently, the focus of the EMPOWER program during this period included an emphasis on providing social and emotional support, and mentors were encouraged to communicate over telephone or online via videoconferencing.

### 2.5. Data Collection

Qualitative data were collected using progress journals and individual semi-structured interviews with mentors. The journal entries were submitted throughout the course of the study by mentors who provided data on progress and successes, challenges and insights about experiences, and general reflections on the mentoring process and outcomes for mentees. Individual interviews with 15 mentors were conducted between June 2020 and January 2022. All the participants had mentored at least one mentee. The triangulation achieved by using multiple methods of data collection provided in-depth information and increased the rigor of the study [42].

### 2.6. Data Analysis

Interviews were transcribed verbatim from audio recordings and the coding of interview transcripts of mentors was conducted independently by two authors using Braun and Clarke’s inductive thematic analysis technique [42], who then compared themes and sub-themes and reached a final consensus through discussions with all authors. Analysis of the qualitative data provided information on the mentors’ experiences, their perceptions of the mechanisms by which the program had been successful, the specific challenges they faced, and their recommendations for future improvements. Initially, 12 codes were identified, and a coding framework was developed. Upon re-reading the transcripts and journal entries and associated literature, the following changes were made: ‘motivated by mentee enthusiasm’ and ‘fulfilling’ were condensed into the ‘Driven by intrinsic motivation’ theme; ‘respect’ and ‘importance of listening’ were initially coded separately but were combined with ‘establishing relationships’ to make ‘building trust and respect’. This was then added to ‘wider connections’ which represented the connections made via the program workshops. Together, these codes became ‘Importance of building connections’. The codes of ‘systemic barriers’, ‘particular disadvantage’ and ‘overcoming inertia’ were combined to make ‘intersectionality’ which was then added to ‘resources for mentors’ to make the theme of ‘The invisible wall’ to reflect how mentors described the combined health, emotional and physical challenges faced by the women. Finally, the codes of ‘continual learning curve’, ‘mentees change direction’ and ‘difficulties deciding on a specific focus’ were combined to make ‘meeting different needs’ which was added to ‘flexibility and perseverance’ to make the theme of ‘Flexibility and commitment’ which were the qualities needed by mentors to meet the frequent changes. Continual review of the coding framework, ensuring the participants’ voices were honored, led to the final themes [43].

## 3. Results

Mentors resided in Australia for an average of 19 years. Over half of the mentors (n = 12) were of South Asian background with mentors also from Southeast Asia, North-central Asia, the Middle East, and Europe. To provide context, demographic details of the mentees are also presented. Half of the mentees have resided in Australia for 0–5 years and the other half for 6–10 or more years. Over half of the mentees were of South Asian (n = 9) or Middle Eastern (n = 8) background with mentees also from other regions of Asia, Africa, and Europe. The top three visa categories that the of mentees arrived in Australia were: Student visa (n = 8), Partner visa (n = 7), and Refugee visa (n = 4) and twenty-five have a tertiary education.

Thematic analysis revealed that overall, mentors believed the program had worked well, with positive outcomes for themselves and the mentees, even if those outcomes were not related to employment. The following five themes pertaining to the mentors’ roles were identified: (1) Driven by intrinsic motivation; (2) Importance of building connections; (3) Expectations; (4) The invisible wall; and (5) Flexibility and commitment (Table 1).

### 3.1. Driven by Intrinsic Motivation

The mentors’ main motivation in joining the program was to share their own experiences and knowledge to help others. They were highly motivated to use their own social and cultural capital to help build that of their mentee(s). They described the process as a continuous learning curve, but the mentees’ enthusiasm made this a very fulfilling experience. Intrinsic motivation was also underpinned by the lived migrant experiences of the mentors which were often similar to that of the mentees.

#### 3.1.1. Voluntary Role

Mentors were not paid for their role, apart from a small honorarium to cover transport costs. This was essentially a volunteer role built on a desire to help others. Mentors felt it was important that mentors’ intentions stemmed from altruism and recommended thorough screening of mentors at recruitment.

“*I wanted to give back. It was good giving back*.”

(Mentor 13)

“*You need to find mentors that actually genuinely care for these people*.”

(Mentor 5)

#### 3.1.2. Fulfilling

The mentors all described the experience as fulfilling and rewarding. There was an element of pride and satisfaction in what they felt had been a positive contribution to creating change. They enjoyed sharing their own employment journeys, especially when they were in the same field.

“*Being able to help and build a transparent, honest relation with my mentee gave me a great satisfaction and offering my story and experience which meant offering insight for my mentee development was a great pleasure*.”

(Mentor 2)

“*It was good just to share my knowledge and my experience of finding work with my mentee. She’s studying along the same sort of course that I have also graduated*.”

(Mentor 15)

“*I feel much fulfilled as I could be a part of the program and empower and mentor a female in supporting she achieve/work through her goals*.”

(Mentor 14)

### 3.2. Importance of Building Connections

Mentors reported that establishing a personal connection with their mentee was a primary initial goal and was made easier by the sharing of their own migration experiences. Undertaking simple activities together in the beginning, such as exploring general likes and dislikes, assisted with this process. Mentors with similar professional backgrounds and cultural backgrounds to their mentees found it easier to build connections and provide support.

“*She was very shy and reserved at our first meeting but when I introduced myself and shared my story and my struggle about setting in Australia then she felt more comfortable and started to talk to me*.”

(Mentor 7)

“*I have been able to work with a mentee who is currently studying in a similar field as myself and I found it quite straightforward to support her*.”

(Mentor 15)

“*It was a wonderful experience for me as a mentor, in particular, we both were from the same cultural background. It makes the match easy and to understand the mentee and provide advice*.”

(Mentor 14)

#### 3.2.1. Building Trust and Respect

Mentors noted the initial hesitation was best overcome through the building of trust. This was achieved by sharing information about themselves and not proceeding too quickly. Mentors were impressed by the strength, energy, and determination of the mentees. Some mentors intended to remain connected with their mentees past the official end of the program to continue to assist them. Mentors also understood that every mentee was unique. Although considered peers through similar lived experiences, not all responses to migration experiences are the same, and mentors noted it was important to respect boundaries when mentees were resistant to certain activities such as community engagement.

“*You have to go gently and go behind that wall and start to develop the relationship. So, I give them information about myself, who I am. They need to know me before they trust me*.”

(Mentor 16)

“*I find my Mentee to be a well-educated, strong woman who has set goals for herself which I am sure she will achieve*.”

(Mentor 4)

“*I was so happy to see her energy, her spark to make a mark for herself*.”

(Mentor 1)

“*I realized that the issues affecting prospective migrants today in a globalized word are not the same that I faced as a young migrant many years ago*.”

(Mentor 13)

“*I decided to respect her boundaries on community engagement. The idea is to support her not pressure her*.”

(Mentor 3)

#### 3.2.2. Wider Connections

The workshops offered by the research team were identified as an effective way of expanding connections for the mentees. Mentors could not often attend the workshops themselves due to work commitments but saw the workshops as augmenting the individual mentoring sessions. Where they could attend, mentors found them valuable networking opportunities for themselves as well.

“*The workshops were wonderful, she loved them and that they were available to her where she could actually go to [venue] and get a lift in to the program. She really loved all of that you know*.”

(Mentor 8)

“*I did like the workshop aspect a lot because then that’s the time you got to meet all the mentors and you got to meet all the mentees as well*.”

(Mentor 6)

### 3.3. Expectations

The expectations of both the mentors and the mentees influenced interactions between the mentors and their respective mentees.

#### Knowing Mentor and Mentee Expectations

Mentors reported feeling a strong responsibility to ensure positive outcomes for their mentee. Most of the time mentors felt they could meet their mentees’ needs and help them work towards goals. It was important to them to be self-directed and independent. However, mentors felt they needed more support to manage expectations at times.

“*I felt a lot of responsibility to help her and I sometimes felt at a loss. Because there were many things that I didn’t know and I felt at times that she expected me to know*.”

(Mentor 3)

Mentors noted that mentees sometimes had high expectations for the type of employment they were seeking and needed encouragement to set more realistic goals. The importance of building and developing smaller goals into greater achievements was also emphasized by the mentors.

“*By this time she was successful in getting a job, not her dream job but a foot in the door nonetheless, and more importantly now understands the life skill of setting small achievable goals for herself*.”

(Mentor 13)

“*Although she mentioned that the pay rate is very low, I encouraged her to think this opportunity as her first step of her career*.”

(Mentor 7)

Mentees sometimes had expectations for support beyond employment skills. For example, mentor support often expanded to include the mentee’s extended family. Both mentors and mentees saw this as an extension of assisting the mentee, and mentors acknowledged the importance of family for the mentees’ resettlement experiences. However, mentors felt that guidelines could be developed to help them set time boundaries.

“*During the next meeting, we discussed about getting some help on how to find jobs for her [family member]*.”

(Mentor 11)

“*The reach of the program can always be a bit wider than you think it can be. So that was really interesting to see and was actually nice. That should be encouraged more in a formal way because then there could be good guidelines around it*.”

(Mentor 9)

### 3.4. The Invisible Wall

Some mentees were managing complex difficulties that functioned as external barriers to full engagement in the program. Mentors described the combination of these challenges as creating an ‘invisible wall’ that precluded engagement.

#### 3.4.1. Intersectionality

Mentors reported that in addition to general fears and doubts surrounding the job search and application process, some mentees were experiencing the intersection of gender, migrant or refugee status and entrenched disadvantage. Aggregated barriers such as poor language skills, a lack of prior education, physical and mental health challenges and cultural expectations all influenced mentees’ capacity to participate. Systemic barriers such as racism were also noted. Minimal financial independence meant developing the skills required for employment such as gaining a driver’s license or enrolling in an educational course were beyond the mentees’ reach. In addition, some mentors described mentees as having an ‘internal wall’ created by a lack of confidence and long-established habits that meant some mentees were reluctant to leave the house.

“*They are so in their home and children, last few years. It’s very hard to break that habit*.”

(Mentor 16)

“*They’re not even given a chance to go in for an interview because I don’t know maybe their degree is something different or like maybe yeah, a lot of them say their name [referring to employers’ negative reactions]*.”

(Mentor 5)

“*We didn’t have any challenges just took a bit of persuasion to apply for the jobs. [mentee] hates applying for jobs because she fears rejection which in turn affects her mental well-being*.”

(Mentor 6)

In addition to the challenges above, which were expected to some degree, some mentees were facing particularly complex situations such as significant health problems, significant domestic life stressors, and managing family crises both in Australia and in their home countries. These situations were confronting for mentors who felt that in these cases the focus on employability was misplaced. There was a call for more robust screening of mentees to identify participants who would most benefit from the focus of this program.

“*My mentee seems to have significant health problems which affect her ability to find a job and which I am finding confronting*.”

(Mentor 13)

“*It would help if there is better screening of who is coming through, but then again I can understand the battle that you guys are fighting as well because getting CALD people involved can also be difficult*.”

(Mentor 5)

The overwhelming nature of some of these challenges resulted in some mentees suffering from a form of inertia, or resistance to engage in recommendations and advice offered, which was frustrating for mentors. They felt that expectations around frequency of communication should be clear from the start.

“*At the beginning it was a bit difficult. Because I felt their resistance*.”

(Mentor 16)

“*Lack of initiative from the mentee and lack of communication was a challenge*.”

(Mentor 1)

“*I found some suitable English courses, but she was hesitant to start them thinking that they are very hard for her and no time to do them as she is busy with household work*.”

(Mentor 11)

#### 3.4.2. Resources for Mentors

Mentors who were trying to assist mentees with particularly challenging situations requested more support. Whilst they felt comfortable with the more general guidance required for building employability skills and understanding Australian workplace culture, they expressed a desire for more time together with each other and the research team to discuss strategies and progress. They felt these meetings could include the mentees, or just be networking events for mentors themselves.

“*A gathering of the mentors, perhaps after working hours, would be really beneficial*.”

(Mentor 10)

“*There should be like an interim meeting which is either online or face-to-face between [the researchers], the mentee and the mentor…to assess goals*.”

(Mentor 3)

The program booklet provided to mentors was considered useful. The booklet included suggested topics for discussion and exploration, along with simple activities for building self-awareness. Whilst mentors utilized the booklet to different degrees, it was useful for them to have a starting point.

“*Because my mentee was already in a job she didn’t necessarily require some of the materials. But yeah I think it, it does give a broad sort of idea of what, what kind of information we can provide to the mentee and how to support them*.”

(Mentor 15)

### 3.5. Flexibility and Commitment

Mentors demonstrated flexibility and commitment to meet the diverse range of needs in the mentee cohort.

#### 3.5.1. Meeting Different Needs

The need to continually learn and adapt to mentees’ needs was sometimes challenging for the mentors. Mentors had to develop knowledge themselves to help their mentee. This included knowledge on recognition of international qualifications or how to access social services. Some mentors had more than one mentee, which proved to be an additional challenge. However, this was mediated when mentees were enthusiastic and keen to learn.

“*We start with something in one meeting and she comes with different ideas in next meeting. It’s hard to guide her when she doesn’t have clear vision about what to do. But I am trying my best to help her choose the right direction for herself*.”

(Mentor 7)

“*Another challenge is the ability to remain focused on the original objectives mutually agreed when we first met*.”

(Mentor 12)

“*Personally, a bit of a challenge at the beginning was having the two mentees at the same time. my two mentees were completely different to each other*.”

(Mentor 9)

#### 3.5.2. Flexibility and Perseverance

Finding time to meet that was mutually convenient was one of the biggest challenges reported by mentors. This meant flexibility was often needed, at times requiring mentors to change work schedules or personal commitments to meet with the mentee. Flexibility in the mode of the mentoring sessions also became a requirement when supporting mentees during difficult life circumstances and during the COVID-19 pandemic where the frequency of telephone and video-conferencing sessions increased. All mentors found in-person meetings better for mentoring than over the phone or online sessions.

“*She has no flexibility about when she can see me, and I’ve really tried, even despite, I’ve tried to change my work schedule to try and work in with her, but it’s not easy*.”

(Mentor 8)

“*The main challenge so far has been getting [mentee] to meet with me more regularly at a mutually convenient location and time*.”

(Mentor 4)

“*Trying to manage the work and the personal life and mentoring, it was quite tricky*.”

(Mentor 15)

*[Meeting] in person it is less challenging as it is easier to overcome the written language barrier, however over the phone it can be a challenge to support her*.”

(Mentor 15)

## 4. Discussion

The EMPOWER program had positive impacts on the migrant and refugee participants in the study. Mentors generally found the experience fulfilling and utilized professional and cultural experiences that allowed them to capably mentor and guide their mentees towards their goals. Mentors found that through sharing their experiences and being transparent, mentees were forthcoming about the obstacles and barriers they perceived regarding the progress in their careers and job-seeking journey.

### 4.1. Community-Based Participatory Approach

The involvement of community organizations that support refugee and migrant women was a central component of this program. The community organizations provided safe venues for workshops, and assisted with the ethical recruitment of mentees, many of whom were vulnerable. They also assisted with finding mentors who met program requirements. Collaboration with community organizations is important when facilitating programs with refugee and migrant groups and helps to ensure cultural safety and acceptability [25,28,32].

The community organizations also helped to identify appropriate mentors. Those mentors, along with representatives from the community organizations, were consulted in the development of the program. When mentors are involved in program planning the parameters are culturally appropriate and the discussion topics are relevant to the mentees’ needs and contexts [27,28,40]. As the program progressed, the flexibility of the participatory approach enabled mentors and researchers to adjust timelines, delivery methods, foci and content in response to participant feedback [27]. Given the range of backgrounds and experiences in the mentee group this flexibility was important.

### 4.2. Peer-Led Model

The findings of this study demonstrate the advantages of the peer-based model of providing guidance and support. This aligns with previous studies on peer mentoring of CALD women which have been shown to build connections and social support and facilitate individual and communal learning in a safe environment [24,25]. Greater involvement in the community enhances a sense of belonging which improves health and wellbeing [34]. The mentors’ ability to use cultural sensitivity and target their advice and support in a culturally appropriate way was critical to the intervention. The mutual exchange of knowledge between women with similar experiences and backgrounds builds trust, self-efficacy, empowerment and confidence, and reduces isolation [28,30,32,40]. When people in difficult situations understand that they are not alone in their experience, resilience is built. Furthermore, strong validating relationships can result [44]. The strength of the relationships formed in this study were impacted to some degree by the matching criteria used to match the mentors and mentees.

### 4.3. Importance of Building Connections

The professional background, the cultural backgrounds and the geographical location of the mentors and mentees formed the basis on which mentors and mentees were matched. It was important for the mentees with qualifications to have mentors with similar industry experience. Similarly, for those without transport it was critical that the mentor was geographically close by to minimize the time taken to travel to meeting points. Lack of accessibility is a known barrier in interventions that support refugees [27,29]. Previous mentoring programs for refugee women have prioritized accessibility, providing mentoring in women’s homes [33] or at venues close to mentees’ homes and communities [25]. However, it must be acknowledged that this can put strain on the mentors who may need to travel themselves to make the sessions possible.

Mentors’ own migration experiences were not always the same as those of their mentee, with different priorities and challenges. This highlights the importance of mentors listening to the mentee and identifying the most relevant issues. It was important that mentors did not presume to know the barriers faced or the specific goals of their mentee based on their own experiences. Their main role was to share and support. Active listening is a key mentoring skill and was prioritized in the mentor training. Active listening can help mentors empathize and reflect [45] and is key to problem-solving and maintaining dignity because it recognizes the communication practices, cultural norms and practices of mentees [46,47]. This ensures culturally responsive support for refugee and migrant groups.

### 4.4. Mentor’s Self-Efficacy

Whilst not measured empirically in this study, mentors demonstrated high levels of self-efficacy and commitment. The range of needs among the mentees was broad, and mentors undertook to gain knowledge of government and employment systems and how to access resources as needed, describing the process as a continual learning curve. Mentors who were committed to learning and were able to adapt to a range of challenges appeared to feel satisfaction in their role. Mentor self-efficacy has been shown to be important in successful mentoring relationships, indicating a greater likelihood of the mentor scheduling regular contact, facilitating knowledge and skill development and persevering through relationship obstacles [48,49]. Mentor self-efficacy has also been linked with mentors’ satisfaction with the mentee relationship [50]. More research is needed on whether mentor self-efficacy can be enhanced through mentor training and supervision [48].

### 4.5. Challenges Faced by Mentors

As mentors are an inherent part of the mentoring process it is necessary to identify and address their challenges so that programs can become sustainable. There were sometimes mismatches in expectations between the mentors and the mentees around goals, time commitment, purpose and possible outcomes of the program. Hird (2021) notes that the expectations of mentors and mentees can be difficult to change [51]. In addition, Egege and Kutieleh (2015) posit that the lack of standard definitions of what constitutes a mentoring program leads to difficulties in creating benchmark practice and guidelines [52]. However, clarification of mentee expectations and motivations at enrolment is important. Some mentees enrolled on the encouragement of a friend but showed less enthusiasm for the mentoring after it commenced. It was difficult for the mentors when their mentee was not committed, and mismatched expectations can lead to attrition [33]. Mentors were also very busy with their own work and family commitments. Time management is one of the limitations of a mentoring system using peer mentors who are working. Mentors also expressed concerns about the scope of their role beyond initial mentee support, including with the mentees’ families.

Extended family support was provided organically by mentors throughout the program. Mentors, to build rapport and trust, did not feel able to refuse when asked to help with non-employment-related family issues. Although parameters were provided through the mentor training, the mentor program manual, and the code of conduct to keep the relationship professional, the mentors found it difficult to set boundaries once immersed in the program. Guidance on setting boundaries, both at the commencement of the program and at interim points would be helpful in future programs. However, it must be acknowledged that for refugee and migrant women, the importance of family is well-recognized and cultural expectations around their role in the family may be strong [10,11]. Given the holistic and community-based nature of mentoring with refugee and migrant women the possibility of helping the extended families of mentees should be an acknowledged part of the program.

Some mentees were experiencing particular difficulties, and mentors struggled to help mentees experiencing entrenched disadvantages. These included legal, financial, physical and mental health challenges. Where possible, mentors were able to develop plans to address some of the issues in small steps. However, this is an area where considerable support for the mentors is required. One area of concern was with mental health consequences from pre-migration trauma. Paloma, de la Morena and López-Torres (2020) and Paloma et al. (2020) report that post-traumatic growth can be improved for refugees through participation in a mentoring program, with improvements in appreciation of life, personal strength, relating to others and recognizing new possibilities [28,40]. By becoming mentors themselves, former refugees can build resilience and empowerment.

Im and Swan (2021) note that trauma-informed care could be useful in addressing the gaps between current mental health care services and the needs of refugees and can also be used with services that offer non-professional psychosocial support [53]. The key consideration is that trauma-informed care needs to be culturally informed and appropriate. Peer mentors from similar cultural backgrounds to the mentees can bring cultural sensitivity and responsiveness to the mentoring relationship. Acknowledging the strengths of cultural capital and the role of families is, in itself, a form of trauma-informed care and can promote resilience and social integration [53]. In this regard, peer mentors have the foundations for delivering trauma-informed psychosocial programs recommended by Im, Rodriguez and Grumbine (2021) and would benefit from specific training and resources to build on these strengths [54].

### 4.6. Strengths and Limitations

The multiple sources of data added to the richness of the data collected. By collecting data in both narrative and interview format, participants could discuss their experiences at different time points and address different aspects of the program, thus enabling researchers to gain a comprehensive understanding of the phenomenon. Data were collected over a six-month period, ensuring prolonged engagement with the data and enhancing the credibility of the study [55].

One limitation was the inability for mentors to provide data anonymously. The mentors’ journal submissions were named and identifiable. The mentors were aware that the research team, who also oversaw the operations of the mentoring program, would be reading the journal entries. There is a possibility that social desirability bias influenced the findings. The mentors may have been reluctant to criticize the program, an issue identified in previous work on mentoring with CALD groups [27]. We recommend that mentors are given opportunities to provide general feedback anonymously so they can be as open as possible.

Another limitation of this study is the small sample size; however, the sample size is typical for qualitative studies, and the findings presented are not intended to be generalizable. The depth of information provided enhances the transferability of the findings.

The original inclusion criteria for the mentoring program needed to be altered part way through the study. Due to, in part, the challenges of COVID-19, it became necessary to include non-refugee migrant women when we experienced difficulties in recruiting and retaining refugee women from different ethnic backgrounds. However, the flexibility accorded to the research team by the community-based participatory approach enabled us to transition to the wider sampling criteria, and to respond to emerging challenges.

### 4.7. Recommendations

The perspectives of the peer mentors from this pilot study indicate some recommendations for future programs utilizing the peer-based model. These include providing trauma-informed training to mentors to complement their existing skills in culture-informed care. Mentors also need resources to help address the many practical, structural, and systemic barriers to health and well-being faced by refugee and migrant women.

Most of the peer mentors in the study had demanding work obligations which on occasion impeded the frequency of support that they could provide to their mentees. To mitigate the problems associated with the time pressures for mentors, future programs should target retired or part-time peer mentors with past professional experience. This may reduce the load on mentors and allow even more flexibility for dyads to meet. It would also improve the ability of the research team to provide mentor support. In the current program, some mentors requested interim support, but as all mentors worked, attendance at any organized meetings was low.

Further consideration could be given to the specific goals and expectations of the mentor and mentees to facilitate their alignment. This could be done at the screening stage where detailed, rather than general, information on career or employment-related goals of mentees and expectations of both mentors and mentees are collected.

Consideration needs to be given to the matching process. Suggestions include a two-hour networking event at the commencement of the program where all mentors and mentees share their experiences and expectations before deciding themselves on the most suitable pairings. However, this method would require all participants to be available, something that is a known difficulty with mentoring programs.

Recommendations for future research include incorporating follow-up with mentees long term to assess the effectiveness of the program and its influence on well-being over time. Future research could also take a different approach in the study design by comparing quantitative and qualitative data collected from mentors who participated in a mentoring program to a control group of comparable participants that previously have no experience or training in peer mentoring. Studies need to also consider larger sample sizes to increase the generalizability/transferability of results.

Future research could also expand the scope of this pilot study by evaluating the effectiveness of a work experience and internship program through engagement and partnerships with small businesses and industry on employment and health and well-being outcomes of this cohort.

## 5. Conclusions

The EMPOWER peer mentoring program aims to enhance employability and networks for vulnerable refugee and migrant women. Mentors were intrinsically motivated to provide culturally informed support, which they found fulfilling. They built strong connections with the mentees, based on trust and respect, and helped the mentees build wider connections. The range of backgrounds and needs among the mentees meant there was a wide variety of expectations, the meeting of which sometimes became problematic. The challenges faced by refugee and migrant women sometimes made it difficult for mentors to gain momentum, but the perseverance of mentors and flexibility in the program enabled mentees to build confidence and self-efficacy. Mentors with lived experience of migration were critical to providing validation and acknowledgment of the mentees’ stories. There is a need for mentors to have training in trauma-informed care to augment their existing skills in culturally responsive care [54]. Regular mentor support is also essential for sustainability and to maintain good outcomes. Peer mentoring programs with refugee and migrant women have the potential to enhance employment and health outcomes in this vulnerable group.

## Figures and Tables

**Table 1:** Mentors’ insights into the Empowerment and Peer Mentoring of Migrant and Refugee Women pilot program (EMPOWER).

Theme	Sub-Theme
Driven by intrinsic motivation	Voluntary role Fulfilling
Importance of building connections	Building trust and respect Wider connections
Expectations	Knowing mentor and mentee expectations
The invisible wall	Intersectionality Resources for mentors
Flexibility and commitment	Meeting different needs Flexibility and perseverance

## Data Availability

The datasets produced and analyzed for this study are available from the corresponding author on reasonable request.
